# Development of a Tablet Computer Application for HIV Testing and Risk History Calendar for Use With Older Africans

**DOI:** 10.3389/frph.2021.671747

**Published:** 2021-12-01

**Authors:** Dilruba Parvin, Abu Saleh Mohammad Mosa, Lucia Knight, Enid J. Schatz

**Affiliations:** ^1^Department of Electrical Engineering and Computer Science, University of Missouri, Columbia, MO, United States; ^2^Department of Health Management and Informatics, University of Missouri, Columbia, MO, United States; ^3^Institute for Data Science and Informatics, University of Missouri, Columbia, MO, United States; ^4^Division of Social and Behavioral Sciences, School of Public Health and Family Medicine, Faculty of Health Sciences, University of Cape Town, Cape Town, South Africa; ^5^School of Public Health, Faculty of Community and Health Sciences, University of Western Cape, Bellville, South Africa; ^6^Department of Public Health, University of Missouri, Columbia, MO, United States; ^7^Medical Research Council/Wits Rural, Public Health and Health Transitions Research Unit, School of Public Health, University of the Witwatersrand, Johannesburg, South Africa

**Keywords:** HIV, NCD, Testing and Risk History Calendar, life history calendar, application

## Abstract

Life history calendars (LHCs) are able to capture large-scale retrospective quantitative data, which can be utilized to learn about transitions of behavior change over time. The Testing and Risk History Calendar (TRHC) is a version of life history calendar (LHC) which correlates critical social, sexual and health variables with the timing of HIV testing. In order to fulfill the need for time-bound data regarding HIV testing and risk of older persons in South Africa, a pilot of the TRHC was performed using a paper fold-out grid format. Though the TRHC study in this format was effective as older persons were able to recall details about their HIV testing and risk contexts, the interview process was tedious as data were collected manually. Development of a tablet application for TRHC study will improve data quality and make data entry and collection more automated. This paper presents the development of the TRHC application prototype in order to collect TRHC data electronically and provides a platform for efficient large-scale life history calendar data collection.

## Introduction

Human immunodeficiency virus (HIV) is a communicable disease which is a major health concern worldwide especially in Africa. With the increasing number of older persons living and becoming infected with HIV, as well as the complexity of health care utilization due to the co-occurrence of HIV and non-communicable diseases (NCDs) epidemics in this population, there is a need for more detailed and time-bound data on older Africans (1–4). HIV prevalence among those 50-plus is as high as 16%, yet reported ever-testing prevalence for HIV among South Africans 50-plus is significantly lower than those 15–49 (54 vs. 78%) (5). Older persons often test at a late stage of HIV-infection when viral load is higher and thus are less responsive to antiretroviral therapy (ART) (6, 7). Over the life course men have lower reported testing rates and test “later” than women (8, 9), but we know little about women's testing behavior beyond child-bearing ages. Many older South Africans do not know their status due to missed opportunities for HIV testing and misunderstood drivers for HIV infection at older ages (10). Key information is needed on social, sexual, and health histories and how these align with HIV testing in order to understand risk in this population.

While most surveys in Africa collect data on HIV testing and recent risk behaviors, the variables are limited and generally collected in cross-sectional survey format, and most often focus on those aged 15–49. New data sources are essential in a setting where the number of people living with HIV (PLWH) at older ages is increasing, due to the scaleup of ART and new infections (11). However, many older persons are not tested routinely for HIV as the focus of *HIV testing* is on people aged 15–49 (12, 13), the age when they are considered sexually active and therefore “at risk” of HIV (14, 15). In this same population, NCDs are more readily recognized as diseases of aging, but diagnosis and management still need additional attention particularly for HIV (16–21).

Life History Calendars (LHCs) have been implemented in the collection of reliable and valid retrospective data, including from older persons and in lower and middle income countries, in the social, behavioral, and health sciences since 1969 (22–24). In the United States the Panel Study of Income Dynamics (PSID) (25) and Health and Retirement Study (HRS) (26) have both made use of this form of data collection. LHC test-retest reliability studies reveal high levels of temporal agreement (between 87 and 97%) in a number of areas, including employment and agricultural practices, with modest loss of agreement for earlier periods of time (27, 28). LHC validation studies that compare agreement with another source of data have reliably produced retrospective reports of higher data quality in comparison to standard questionnaires among a wide range of measures including relationships, cohabitation, health status, disability, and labor histories (25, 29, 30). Better data quality in LHCs has been traced to the higher prevalence in the use of sequential (e.g., remembering one's earlier and later partners) and parallel (e.g., remembering one's health status during a relationship with a specific partner) cues (31–33). LHCs have led to important scientific inferences regarding the interrelationships among life course events, including how life course exposures impact later life health and well-being (34–38). The current format of LHC data collection, however, is either a telephone interview or a large fold-out paper grid.

In 2018, we piloted a Testing and Risk History Calendar (TRHC) instrument using a paper fold-out grid (1). The TRHC is a Life History Calendar (LHC) which identifies the relationship between risk and health for a certain period of time for South Africans (over 60). TRHC enables us to collect empirical data about the HIV testing and social, sexual, and health risk histories of older urban South African (1). The TRHC captures individuals' transition from middle to older ages. The format of the instrument allows for the collection of more in-depth social context data related to testing decisions, and more detailed social, sexual, and health timelines, than are available from standard cross-sectional data (37). The hybrid (quantitative-qualitative) nature of the instrument and interview encourages commentary by the respondent and interviewer that provide context for understanding the relationship between timelines (1). For example, with the TRHC, we will be able to explore whether there are missed testing opportunities, i.e., older persons who was diagnosed with an NCD and engaged in care on a particular date, but not tested routinely for HIV on or after the NCD diagnosis. Further, the TRHC instrument could be adapted for use with other older African populations, as well as for use in younger populations, to enable the collection of retrospective data about testing and risk histories.

The paper fold-out grid format is cumbersome for the interviewer to collect data (1). Development of a tablet application will make the data collection process feasible for the interviewer. Additionally, questions with multiple choice options are not feasible to show in paper format and are more suitably assessed using a pop-up window when data is entered for a particular field. Besides, entering participant comments in a large-scale study would not be feasible by hand, but as part of the application can be incorporated as “margin comments” and make data readily available in an online database minimizing need for data entry and management. Developing a tablet application with customizable software to collect the data, will revolutionize LHC data collection in large populations, and for projects similar to the TRHC. In order to attain this purpose, we have developed a prototype of the TRHC application to support android devices (specially tablet). The purpose of developing the TRHC application is to incorporate the functionality and overcome the challenges associated with the paper fold-out grid format.

This paper aims to illustrate the development of an interviewer administered tablet application which allows for the collection of calendar format TRHC data and associated survey data simultaneously. The method of developing the application is described in section Method. Section TRHC Application Workflow, Design, and Features showcases the workflow, design, and features of the application. Finally, a discussion section has been added to section Discussion with concluding remarks in section Conclusion.

## Methods

The TRHC application contains a calendar format as well as questions asked through several surveys related to the TRHC study. These surveys include questions related to socio-demographics, relationships, and health about a particular respondent. Each survey is presented in the calendar view format to collect retrospective qualitative and quantitative data about the respondent which helps to understand the relationship between timelines. In the following sub-sections, the data domains related to this study, platform to develop the application to conduct the study and the architecture of the developed application will be described.

### TRHC Data Domain

TRHC consists of three data domains that are closely related to the respondent's health such as: (a) Social Risk, (b) Sexual Risk, and (c) Health Risk.

#### Social Risk

As individuals age, their relationship to sexual risk and access to care shift. Better understanding of the life course factors that impact decisions about sexual relationships, HIV testing, and engagement with care provide potential intervention points for increasing HIV testing and care. Capturing the dynamism and temporal nature of living arrangements, marital status, access to routine care, and pension receipt, are essential to understanding the context in which risk and testing decisions occur. Thus, information about social, demographic, and economic that might impact risk and testing will be collected in the study. Table 1 illustrates a portion of questions related to the socio-demographic activities of a respondent. Information related to the location of the respondent, housing type, marital status etc. are collected in this section. Each question has several selection options with specific coding such as marital status is captured with eight options (see the options and corresponding coding in Table 1).

**Table 1:** Questionnaire related to socio-demographic behavior.

**SI**	**Questions**	**Options**
**Socio-demographics**		
1.	Respondent age	_________
2.	Location	1 Khayelitsha (K) 2 Langa (L) 3 Mitchell's Plain (MP) 4 Cape Town (CT) 5 Eastern Cape (EC) 6 Other Western Cape (WC) 7 Other (O) (specify) _________ 88 Don't know (DK) 99 No response (NP)
3.	Urban/Rural	0 Rural (R) 1 Urban (U) 88 Don't know (DK) 99 No response (NR)
4.	Housing type	1 Formal (F) 2 Informal (I) 3 Other (O)(specify) _________ 88 Don't know (DK) 99 No response (NR)
5.	Marital status	1 Married (M) 2 Divorced/separated (D) 3 Widowed (W) 4 Not in partnership (N) 5 Co-habiting (Co) 6 Other (O)(specify) _______ 88 Don't know (DK) 99 No response (NR)
6.	Economic activity	1 Unemployed looking for work (UL) 2 Not looking for work (NL) 3 Self-employed, regular income (SR) 4 Self-employed, irregular income (SI) 5 Employed, regular income (ER) 6 Employed, irregular income (EI) Other (O) (specify) _________ 88 Don't know (DK) 99 No response (NR)
7.	Pension/Grants	0 No (not recorded on calendar) 1 Yes, old age pension (OA) 2 Yes, disability grant (DG) 3 Yes, work pension (WP) 88 Don't know (DK) 99 No response (NR)

#### Sexual Risk

Despite evidence suggesting that sexual activity and risky behavior continue into older ages (39–41), neither older persons nor providers view older persons as “at risk” for HIV (42, 43). This impacts the likelihood of testing and hides potentially risky behavior (42, 44). The proximity of sexual risk to testing decisions is a key missing piece to understanding older persons' testing decision making, and a potential intervention point for increasing testing among older persons, particularly those with “risky behavior.”

For each romantic or sexual relationship over the past 10 years, relationship dimensions and sexual risk behaviors specific to that partnership will be recorded in this study. Table 2 demonstrates few questions related to the relationship of a respondent. Partners age, date of birth, initials need to be collected. From Table 2, it can be seen that partners' education level has several options such as no schooling, matric, some university etc. which are related to codes 0, 13, and 14, respectively. By addressing these questions, the reasons behind the sexual risk to the participant are anticipated.

**Table 2:** Questionnaire related to Relationship behavior.

**SI**	**Questions**	**Options**
**Relationship**		
1.	Partner's initials	_______ Initials
2.	Partner's age at relationship start	_______Age 88 Don't know 99 No response
3.	Partner's date of birth	______M 88 Don't know 99 No response ______Y 8888 Don't know 9999 No response
4.	Partner's education level	0 No schooling (None) Stand. 1 2 3 4 5 6 7 8 (S1-8) Form 9 10 11 12 (F1-4) 13 Matric (M) 14 Some university (SU) 15 Completed university (CU) 16 Vocational training (VT) 17 Post-graduate (PG) 18 Other (specify) _________ 88 Don't know 99 No response
5.	Year of relationship start	_________ YR 8888 Don't know 9999 No response
6.	Reason for end	_________
7.	Relationship duration	Started before 2010, ongoing (O) ________Start month/year (SM) 88 Don't know 99 No response ________ End month/year (EM) 88 Don't know 99 No response
8.	Partner's residence	1 Same household (SH) 2 Same area (SA) 3 Other urban area (OU) 4 Other rural area (OR) 88 Don't know (DK) 99 No response (NR)
9.	Partner's economic activity	1 Unemployed looking for work (UL) 2 Not looking for work (NL) 3 Self-employed, regular income (SR) 4 Self-employed, irregular income (SI) 5 Employed, regular income (ER) 6 Employed, irregular income (EI) Other (O) (specify) _________ 88 Don't know (DK) 99 No response (NR)
10.	Pension/Grant	0 No (not recorded on calendar) 1 Yes, old age pension (OA) 2 Yes, disability grant (DG) 3 Yes, work pension (WP) 88 Don't know (DK) 99 No response (NR)
11.	Type of relationship	1 Spouse/living as married (M) 2 Fiancé/ promised to marry (F) 3 Serious partner (SP) 4 Dating (D) 5 Casual partner (CP) 6 CSW/client (CSW)
		One-night stand (ONS) Stranger (Str) 9 Other (O) (specify)________
12.	Main reason	1 Married (M) 2 Social pressure (SP) 3 Wanted a partner/relationship/ companion (WP) 4 Sex (S) 5 Physically attracted to him/her (A) 6 Liked his/her personality (P) In love (L) Money/gifts/assistance (G) Convenience (C) Adventure/experimental/fun (F) Didn't want AIDS (DW) Forced/coerced (FC) Other (O) (specify) _________ No reason (NR) 88 Don't know (DK) 99 No response (NR)
13.	Secondary reason	1 Married (M) 2 Social pressure (SP) 3 Wanted a partner/relationship/companion (WP) 4 Sex (S) 5 Physically attracted to him/her (A) 6 Liked his/her personality (P) In love (L) Money/gifts/assistance (G) Convenience (C) Adventure/experimental/fun (F) Didn't want AIDS (DW) Forced/coerced (FC) Other (O) (specify) _________ No reason (NR) 88 Don't know (DK) 99 No response (NR)
14.	Frequency of sex	1 Nearly daily/Daily (D) 2 More than 1 × per week (MW) 3 About 1 × per week (AW) 4 1–2 × per month (AM) 5 <1 × p/month (having sex) (LM) 6 No sex (NS) 88 Don't know (DK) 99 No response (NR)
15.	Contraception	0 None (N) 1 Pill (P) 2 Male condom (C) 3 Female condom (FC) 4 Injection/Depo (ID) 5 IUD (IUD) 6 Implants/Norplant (IN) Diaphragm (D) Vasectomy (V) Tubal ligation (TL) Rhythm/natural (RN) Withdrawal (W) Morning-after pill (EC) Foam/jelly (FJ) Other traditional methods (TM)
		Unknown method (for male Rs) (U) N/A P/R pregnant (Preg) N/A menopause (Meno) Other (O) (specify) ________ 88 Don't know (DK) 99 No response (NR)
16.	Condom use	0 N/A (no sex) 1 Always 2 Most of the time 3 Sometimes 4 Very rarely 5 Never 88 Don't know 99 No response

#### Health Risk

The high prevalence of NCDs in the population introduces complex issues related to the timing of symptoms, diagnoses, treatment and engagement with the health system (16, 45–48). Further, despite current guidelines, NCD and HIV care remain siloed, and once diagnosed with an NCD, the likelihood of an older person getting routine testing for HIV is low (17). Being able to assess missed opportunities for HIV and NCD testing and treatment through both biomarkers at the time of the study and reported NCD/HIV diagnoses and treatment over the recent life course will suggest other key intervention points to improve health and care.

This section of the study captures changes in self-rated health status, as well as NCD screening/diagnosis and management (e.g., hypertension and diabetes; Table 3). Questions related to each change in health status will be collected alongside data about changes in engagement with the health system and access to treatment to enable us to track how these changes correspond to HIV testing. Survey questions focusing on changes in lifestyle related risk will be collected in connection to particular diagnoses. In addition, information about current NCD risk factors will also be collected.

**Table 3:** Questionnaire related to health behavior.

**SI**	**Questions**
**Health**	
1.	Respondent age
2.	Location
3.	Urban/Rural
4.	Housing type

### Application Development Platform

The TRHC application prototype was built for android platform. Android, an open-source architecture was developed by Google LLC and is generally used to create user friendly applications. It includes Operating System, Application Framework, Linux Kernel, middleware, and application which incorporates a set of API libraries for writing mobile applications. Android Software Development Kit (Android SDK) and Java Development Kit (JDK) are used to develop applications. The ability to use Java IDEs/Android Java Libraries makes the application development environment more convenient. The Android Studio Platform can be used to create mobile applications. In this program “Pixel C API 23 (Android 6.0, API 23)” was used as the device.

The TRHC application prototype was developed using ResearchStack (49). ResearchStack is a framework which has been built to make research studies more convenient for the application developers of the Android Platform. It is open source and can be installed by cloning the GitHub repository. JSON and HTML files can be used to build the applications. There are three customizable modules: surveys, consent, and active tasks in ResearchStack. New modules can be built to satisfy the requirements of a survey. Surveys can be built by specifying the questions and types of answers. Taking consent from the study participant is essential to clarify the type of information to be collected from the participant and to declare the scope of information sharing. Active tasks can be added in order to collect more data in which active participation of the customer is required. It allows users to perform activities and data is collected from the sensors of the device such as recoding an audio signal using a device.

### Application Architecture

**Figure 1:**
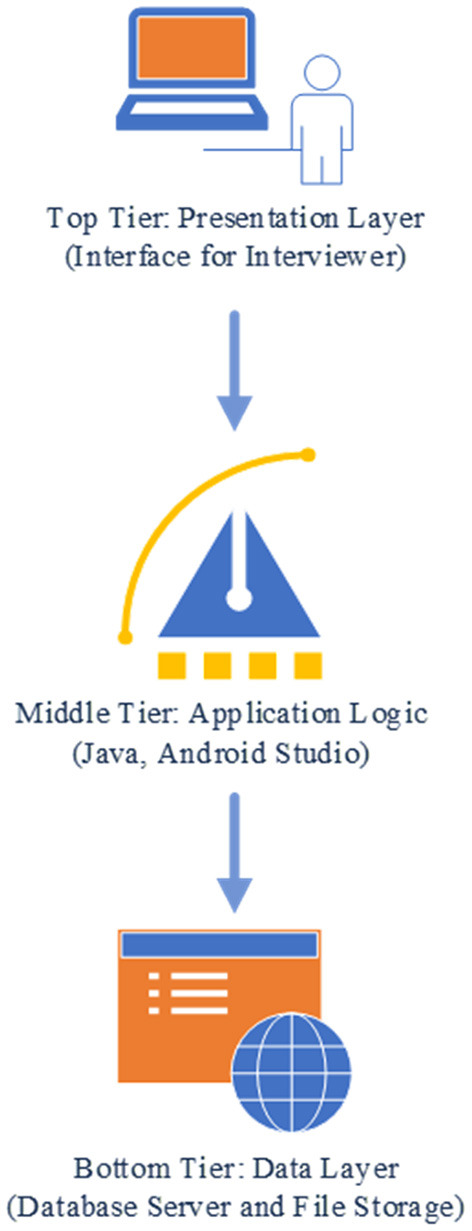
Application architecture.

The preliminary application prototype follows a three-tier structure (Figure 1): top (presentation), middle (application logic), and bottom tier (data). For the presentation tier the interviewer interface was developed. The interviewer interface is accessed through an application in an Android tablet computer, with the potential to create or modify an interview record. While the digitized application enhances the interview capacity, it precludes the benefits of the visual nature fold-out grid calendar. We will assess during pretesting the necessity and possibility of a visual experience for the respondent. The interface for interviewer data entry has been developed using a grid-based presentation layout to accommodate the time dimension in columns and the questionnaire in rows. The questionnaire is grouped in one or more thematic axes (sections) representing the key study domains. The reference period at the time dimension have been divided into smaller time units, such as years or months. Reference points have been added for temporal anchoring points or framing cues. The columns related to the time dimension follows the survey questions toward the left side of the screen and is scrollable by using the two-finger swipe gesture. Each cell of the grid has an option of free text comment entry by using the double-tap gesture (e.g., as “margin comments”). In each cell corresponding to a specific event, a list of multiple-choice (single selection/multiple selection) options is displayed using the single tap gesture. This eliminates the need for memorizing or searching the codes in a data dictionary during the interview. After selection of an option choice, a value or icon is displayed in the cell.

## TRHC Application Workflow, Design, and Features

In the development phase, the goal was to build an application which makes interview process feasible and store the data to a database. To fulfill that purpose, the findings from the pilot study such as contents and features were included while building the application.

**Figure 2:**
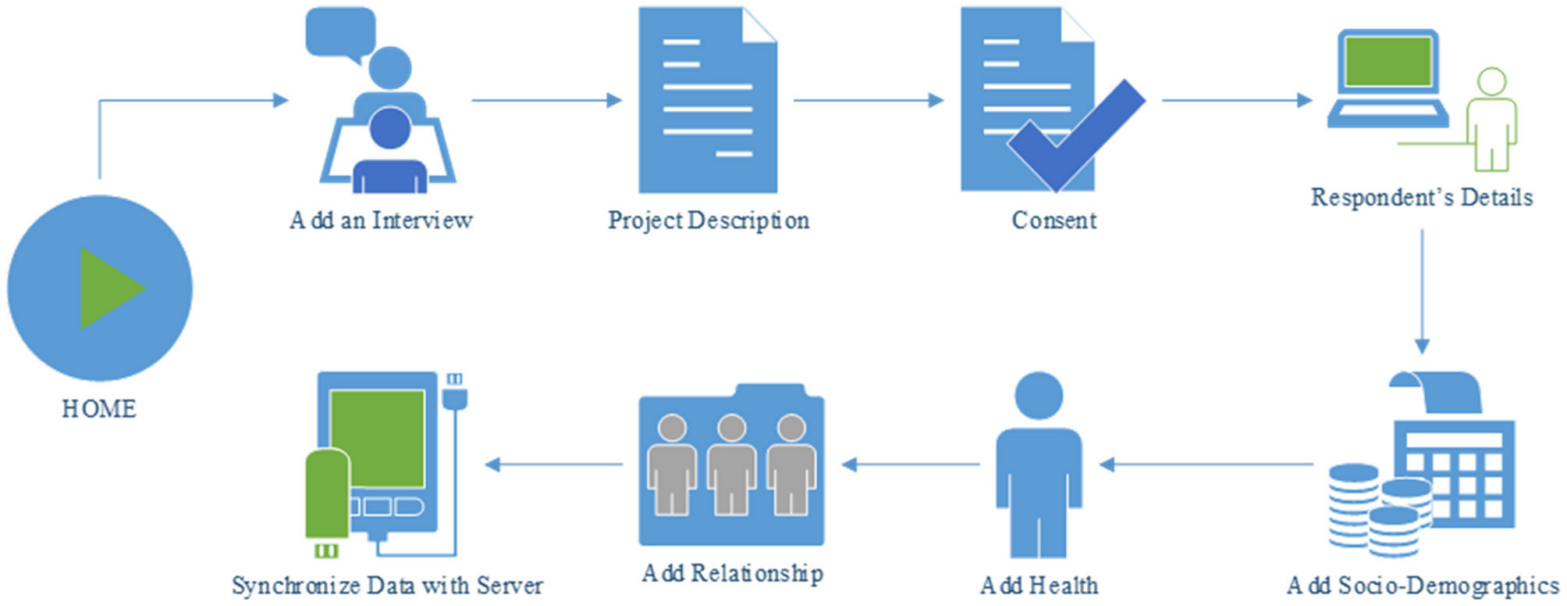
A typical workflow of the interview process using TRHC app.

The first section of the TRHC application is the HOME activity (Figure 2). The read-text-aloud feature of the application can be used to play the overview description of the interview process. A consent section has been added which includes the terms and conditions that needs to be agreed upon by the respondent to participate in the interview. The first part of the interview session is the Respondent's Details where information about the respondent is collected. Surveys for the relevant sub-sections (socio-demographics, health, and relationship) have also been included to the app. The data which are collected during the interview process is stored in a database.

**Figure 3:**
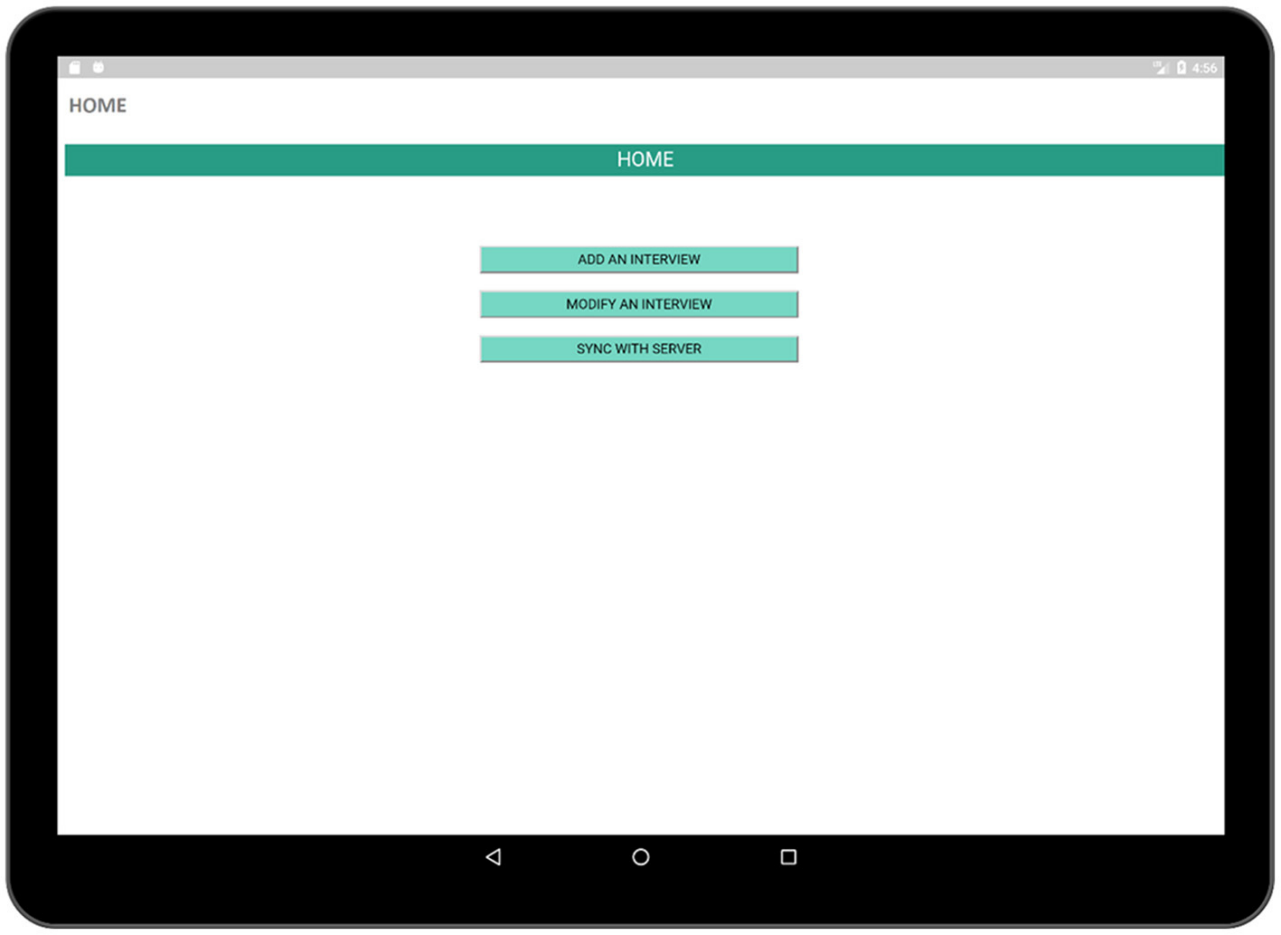
Home activity (contains a list of tasks performed using the app).

We implemented a prototype of the proposed TRHC application to support the requirements described in the previous section. The user interface (UI) designed for this application was simple. The pages of an Android application are generally displayed using activities. The interfaces of an Android application are contained by activities. UI elements are viewed via activities and there is a set of lifecycle methods in order to define the actions and events which are connected via UI elements. Every Android application needs to have at least one activity. Our main activity is the HOME activity (Figure 3) which is linked to several other activities. The primary requirement of the application is a main page in order to display major features available in the application such as creating an interview page in order to conduct an interview with a respondent, modifying a previously executed interview and finally synchronizing the collected data with the database.

**Figure 4:**
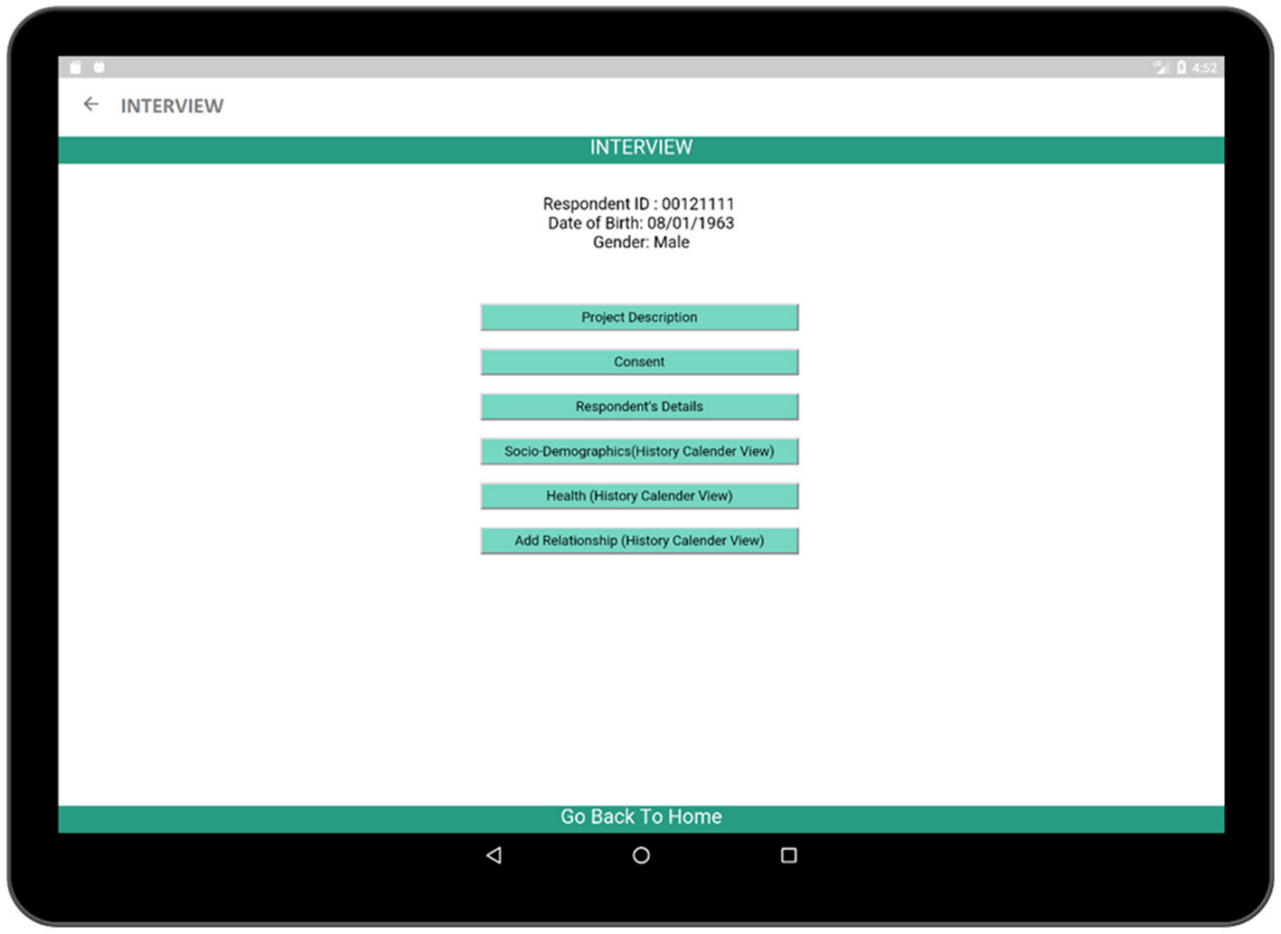
INTERVIEW activity (contains the necessary steps which are necessary to complete the survey).

The INTERVIEW activity (Figure 4) contains the steps necessary for the completion of the interview process. Six activities such as: (a) Project Description, (b) Consent, (c) Respondent's Details, (d) Socio-Demographics (History Calendar View), (e) Health (History Calendar View), and (f) Add Relationship (History Calendar View) are linked to this page and each of them can be launched by using the buttons associated with them. The project description activity contains a brief description of the survey in order to inform the respondent about the interview process. The consent activity is a multi-page activity in which information about data gathering, time required to complete the survey etc. are provided to the respondent so that the respondent is well-informed about the crucial information required in the interview and can provide consent. The Respondent's Details activity contains several sectors in which the respondent can provide personal information such as name, age, date of birth, and gender. Besides, there are three activities related to survey such as Socio-Demographics, Health, and Relationship which are the major parts of the application.

The Project Description activity contains the information related to the interview process as well as brief descriptions of the steps of the interview so that the respondent has a complete idea about the extent of involvement required in the interview process. A read-text-aloud button has been added by launching which the application will read the project description aloud to the respondent making the application suitable to use.

**Figure 5:**
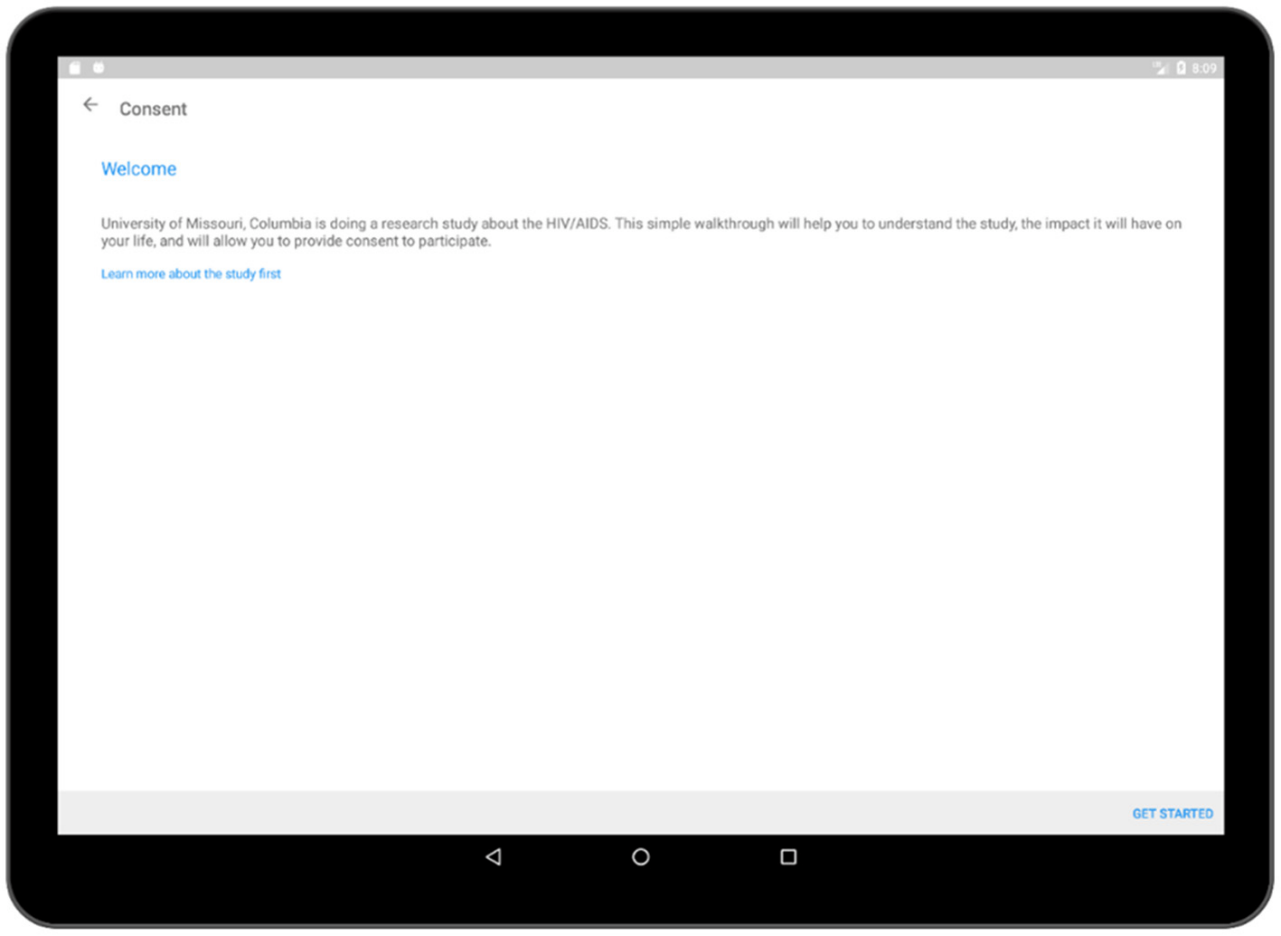
Consent activity (welcome).

In the consent activity (Figure 5), crucial information regarding the survey process such as confidentiality of the collected data, details of the association conducting the survey have been provided. After the respondent has gone through all of the pages related to the consent, he/she is required to provide an electronic signature in the application which will be recorded and stored in the database. More pages related to the consent activity is added to Supplementary Figures 1–3.

**Figure 6:**
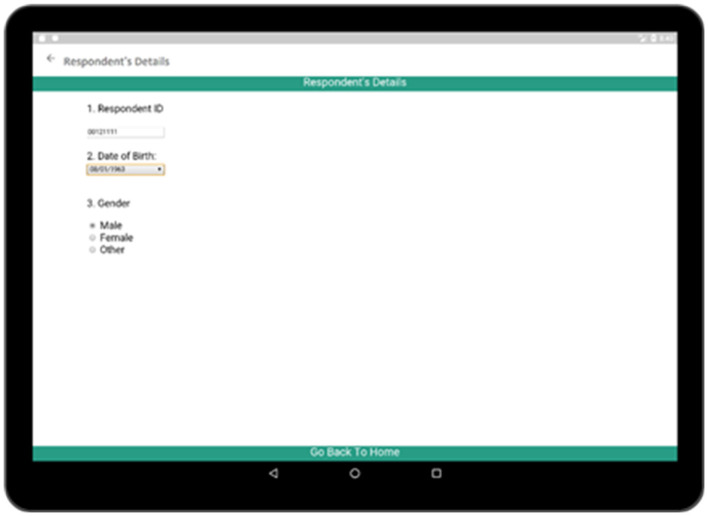
Respondent's details activity (contains necessary information about the respondent).

Respondent's Details activity (Figure 6) comprises of the personal information of a participant. Each participant is provided with a unique identification number during the interview process by which he/she can be identified. The participant is also required to provide his/her name, date of birth, and gender. This information will also be linked with the other activities so that they are also available in other activities to maintain consistency of information.

**Figure 7:**
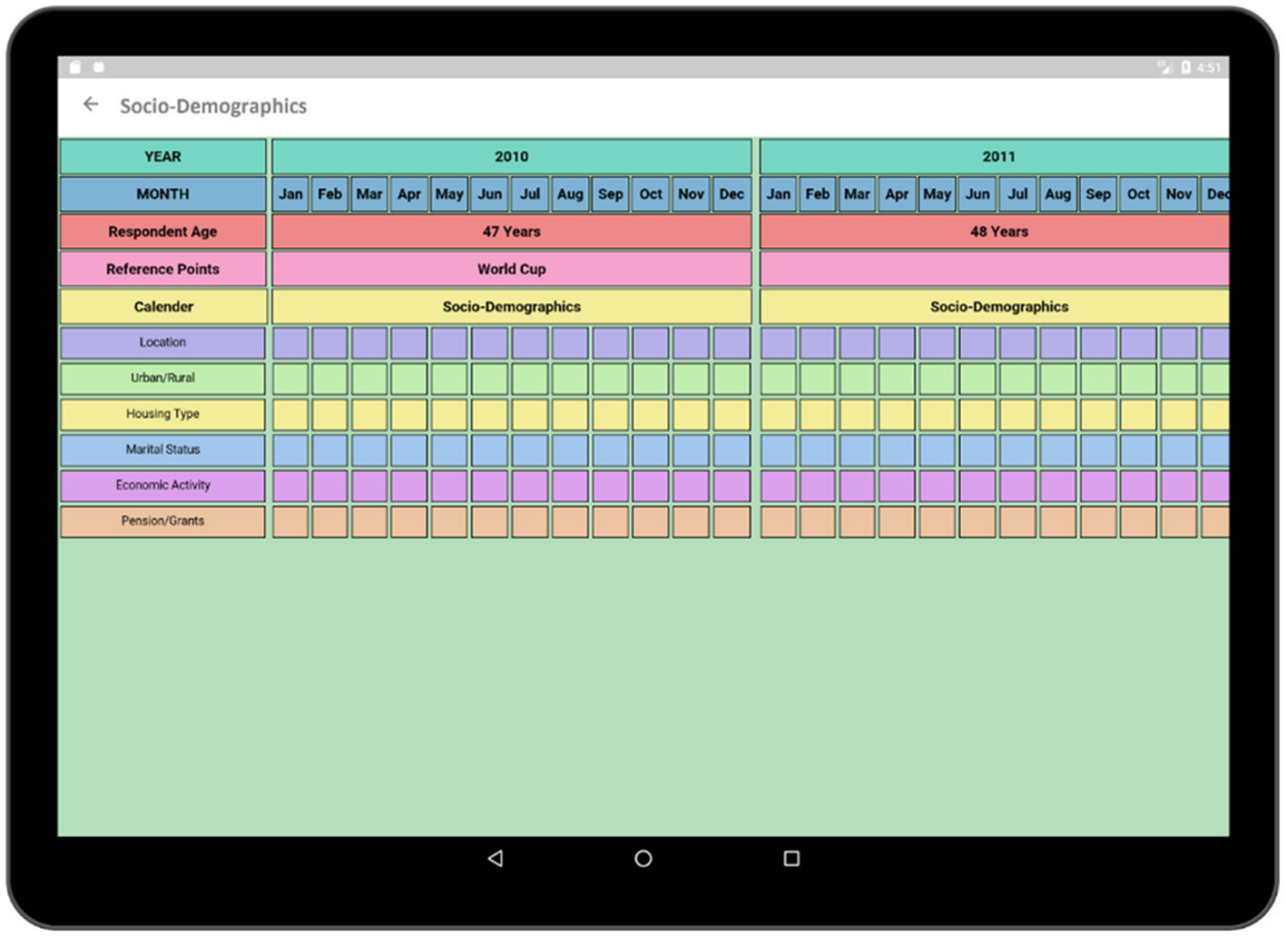
Socio-demographics (calendar view).

The socio-demographic activity contains a survey regarding the social, demographic, and economic conditions of the respondent. The survey format follows similar paper grid-out calendar view format associated with a particular time-span (e.g., 10-years) has been used in an earlier experiment (1). Top rows of the survey contain timeline such as months and years similar to typical life history calendars. Participant's age is also displayed along with the timeline in order to make it easier to relate the age of the respondent to the occurrence of a particular event. The respondent is asked questions regarding area of accommodation, relationship status, income status along with duration (Figure 7). Each sector has multiple choice options related to the timeline which is incorporated in the form of pop-up window.

**Figure 8:**
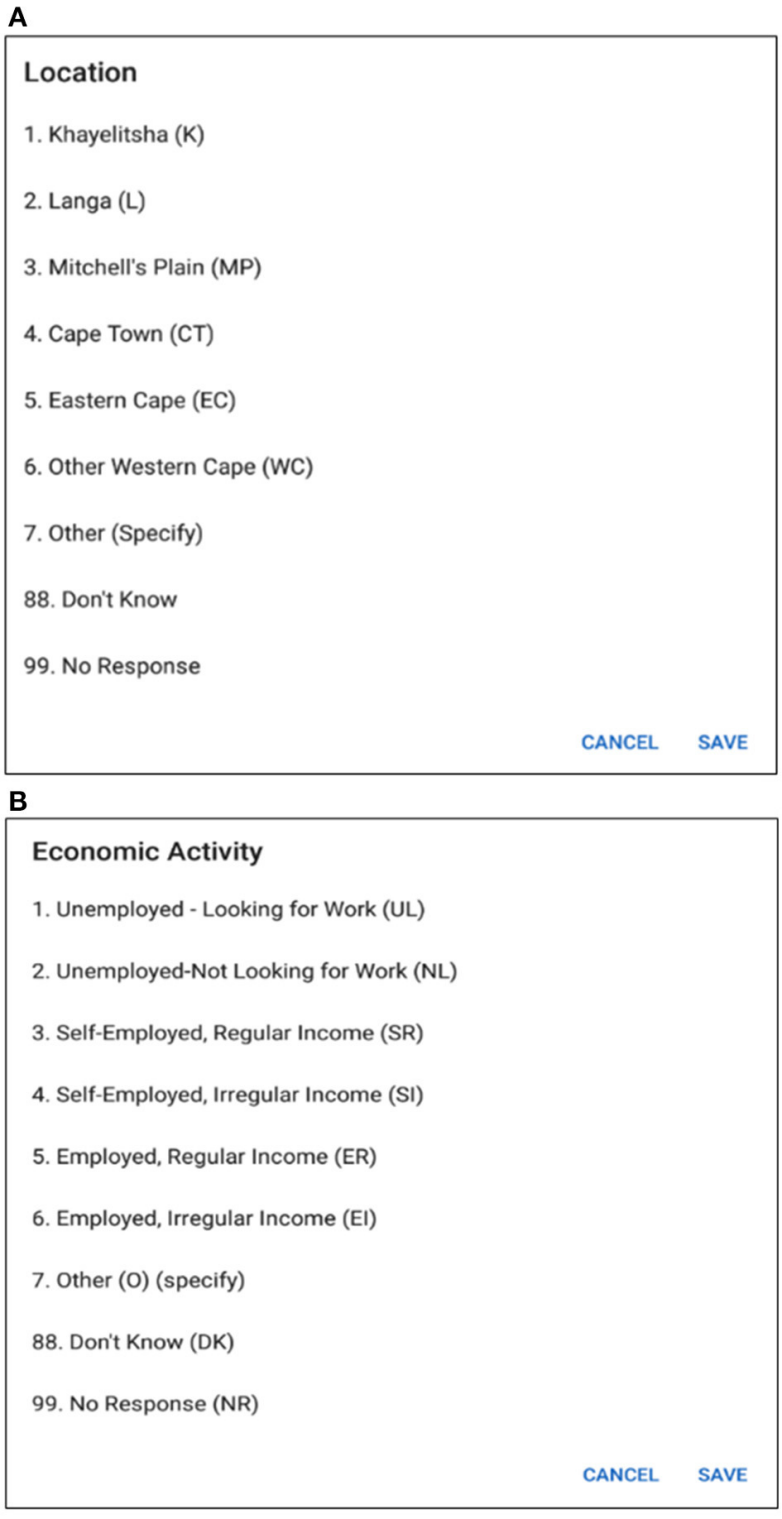
**(A)** Options for location. **(B)** Options for economic activity.

In Figure 8, multiple choice options related to location and economic activity has been displayed. The options of different possible locations are integrated with individual codes such as Khayelitsha, Cape Town and Don't Know are related to code 1, 4, and 88, respectively. The answer can be selected by using a single tap gesture. After the selection of an option, the code of the corresponding option gets saved for that particular time cell. A time duration can also be selected in order to capture the time frame related to the same option and corresponding answer code will be saved for the selected timeline. In this way, the tedious process of memorizing and recording the codes individually related to the options in the paper grid-out is automated via this application. Besides, significant historical events such as the Football World Cup which was hosted in South Africa in 2010 is provided along with the timeline in order to help the participant recall the events related to that reference point which has already proven effective during the paper fold-out interview process.

**Figure 9:**
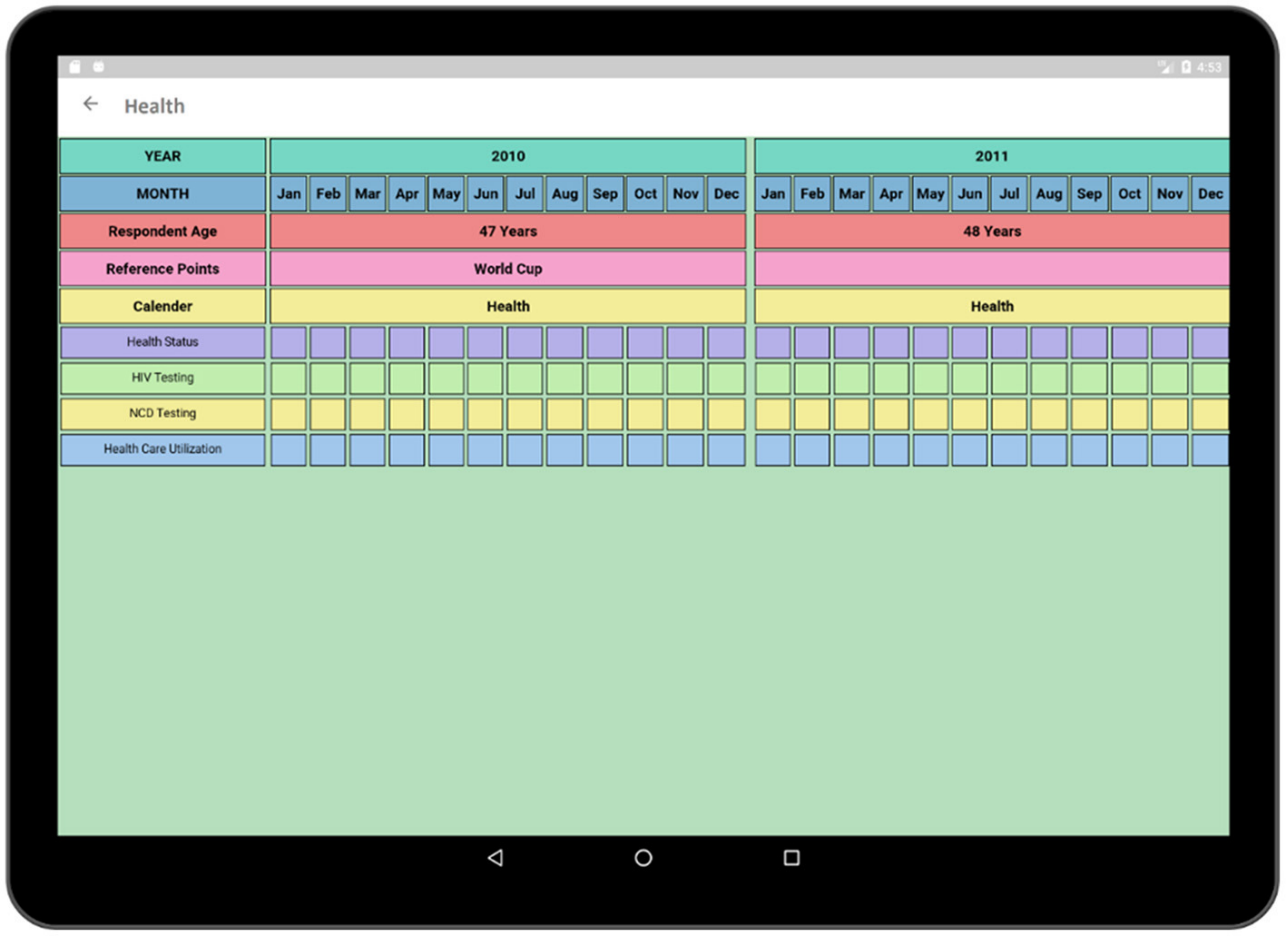
Health activity (calendar view).

The health activity (Figure 9) contains a survey format similar to the Socio-Demographic activity and is one of the most crucial parts of the interview as it contains the survey related to the health conditions and the health care access of the participant. Questions regarding health-related decisions such as HIV testing.

NCD screening are also being incorporated in this section as our purpose is to relate the concurrent information regarding the participant's symptoms, health care usage, and access against a timeline and to find out whether these factors impact on testing decisions.

**Figure 10:**
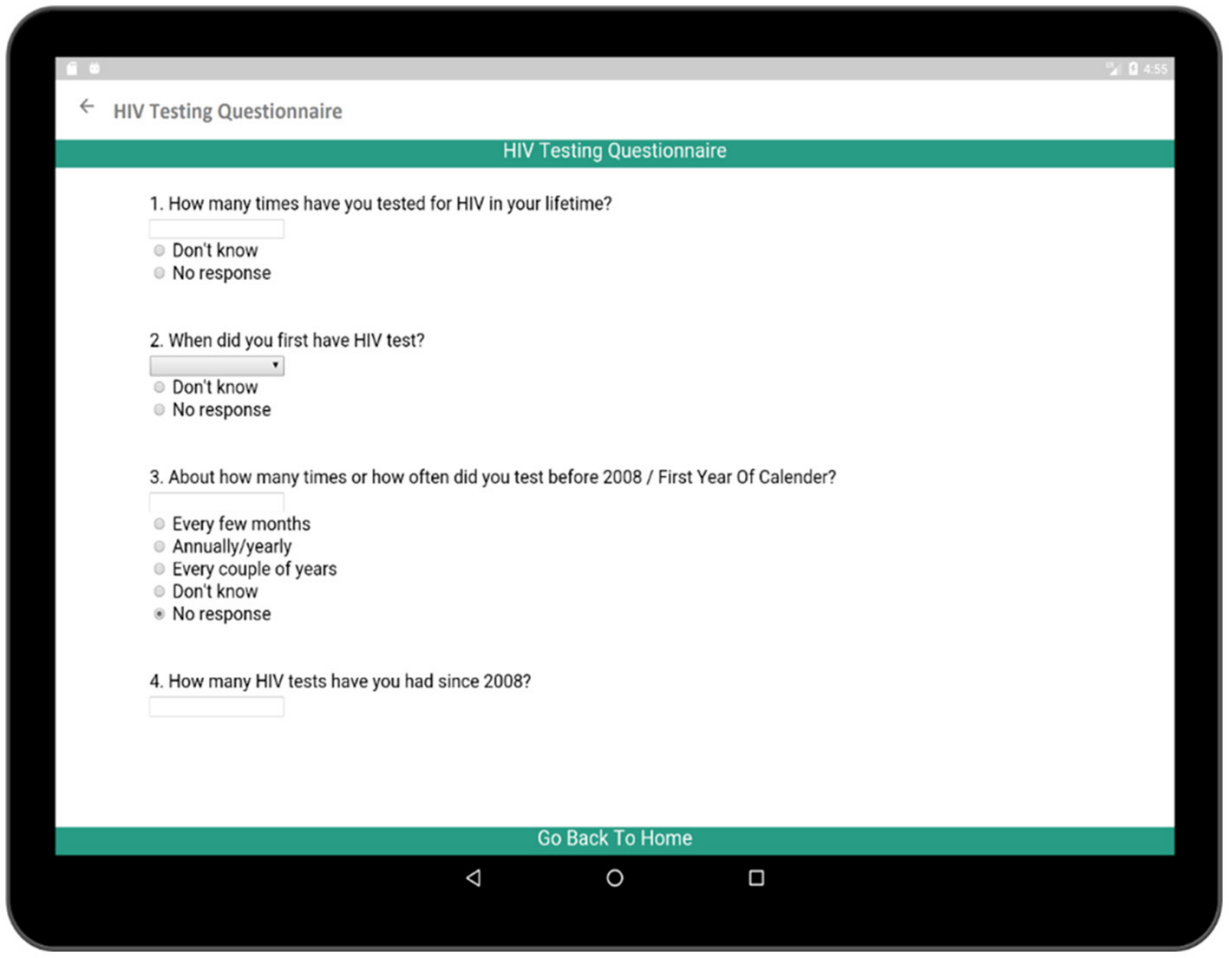
Questionnaire for overall HIV testing behavior.

**Figure 11:**
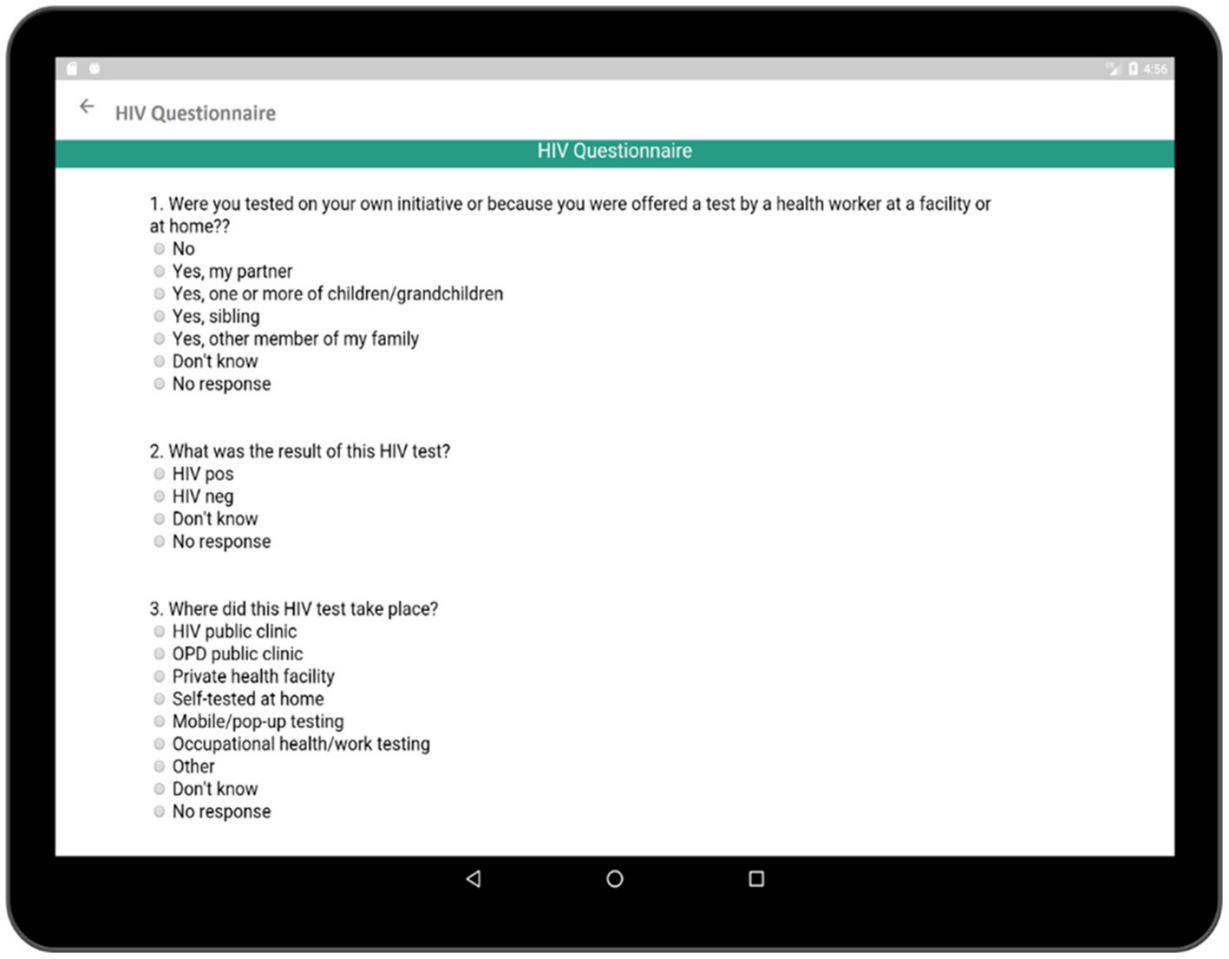
Questionnaire related to recording longitudinal pattern of HIV testing behavior.

The HIV testing section in this survey is a hyperlink which includes general questions as presented in Figure 10. These events reveal important information regarding the patient's health and history. Each time cell also corresponds to a set of questions related to the individual HIV test (Figure 11). The questionnaire collects information regarding the test result, place of the test, test initiative in order to relate this information to patient's current health condition. Both general questions related to HIV and questions related to individual HIV test are added to Supplementary Tables 1, 2, respectively.

**Figure 12:**
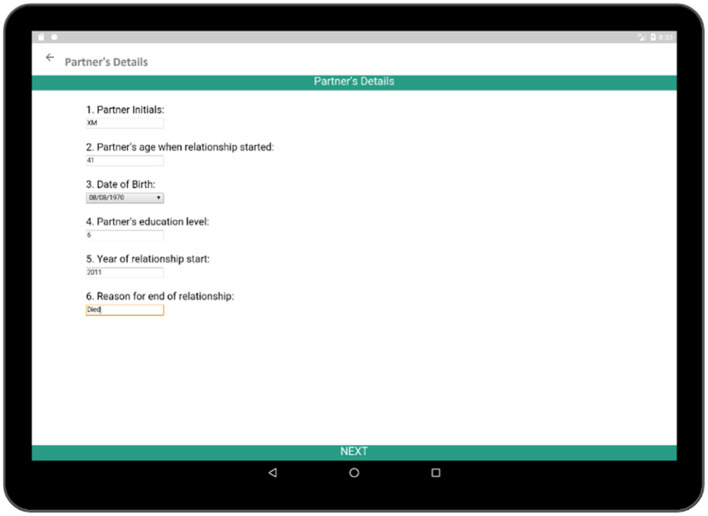
Partner's details (collects some information about the partner).

**Figure 13:**
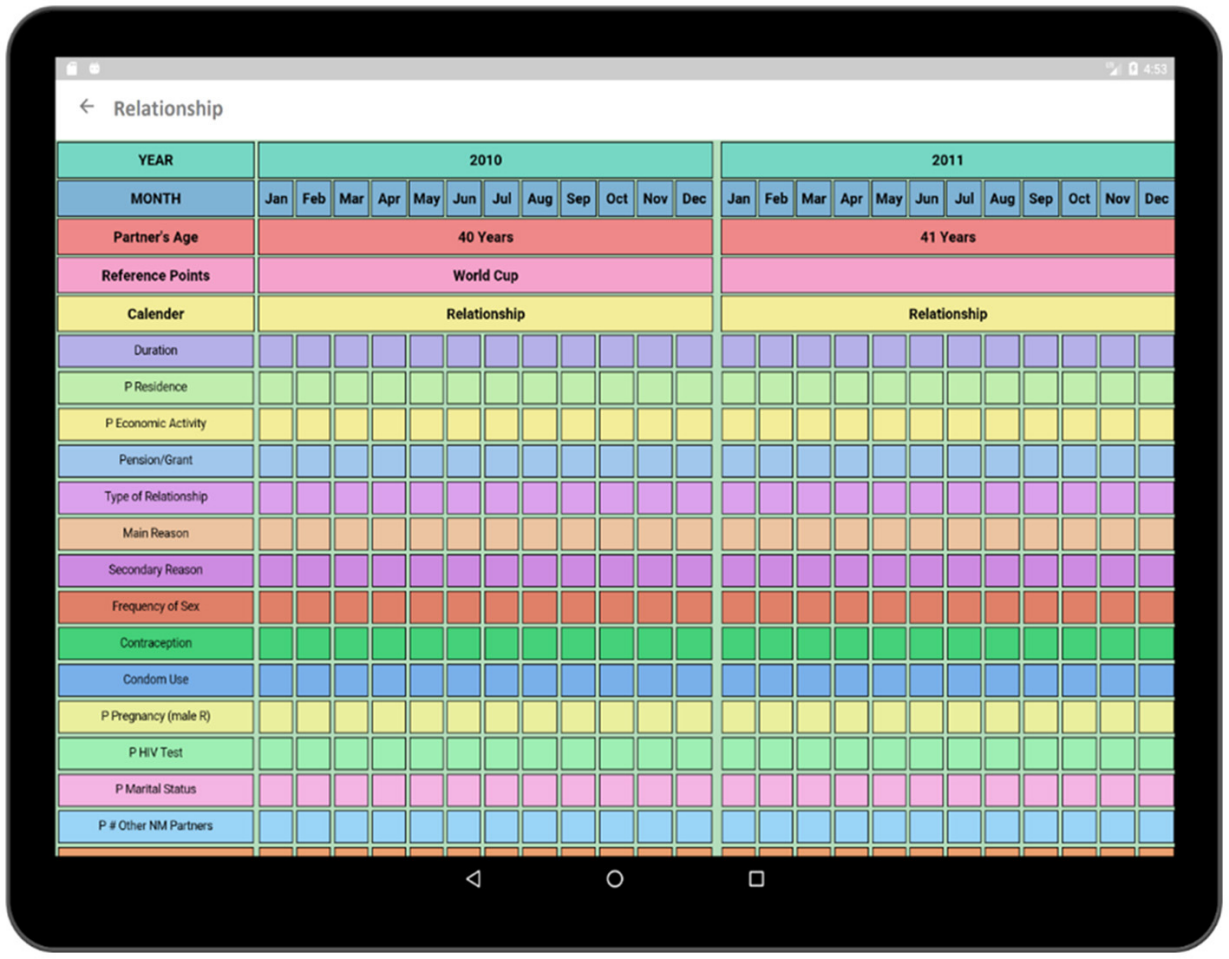
Add relationship (calendar view).

In addition to collecting information regarding the social, economic, and heath histories of respondents, it is also necessary to know about the relationship of the respondent because relationship status is directly related to the sexual and reproductive health of the respondent. Each sexual partner's name, age at the beginning of the relationship, date of birth, education level, relationship duration, and reason behind ending the relationship are collected via the Partner's Details activity (Figure 12). After the completion of the basic information for the corresponding partner, detailed information regarding that relationship is collected via the Relationship survey (Figure 13). The Relationship survey collects information regarding the partner's residence, economic activity, number of non-marital partners, marital status, HIV test result, as these play important roles in the life of the participant. If a participant had multiple partners, this whole process needs to be completed multiple times.

**Figure 14:**
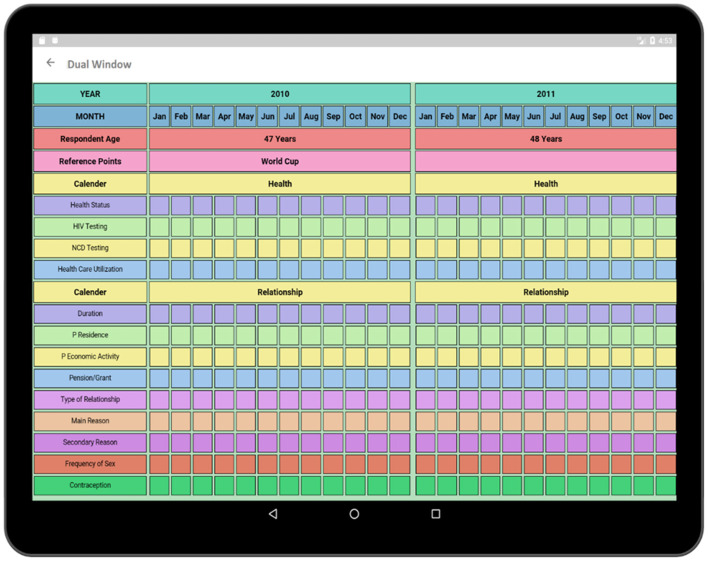
Dual window (compares two surveys to find out the internal relationship between two sectors).

The Dual-Window activity has been created to compare two surveys (Figure 14). The surveys can be selected to be viewed in the Dual-Window activity. The top window is a display screen (Health), and the bottom window is an active window (Relationship). In this section, Health of the respondent is being compared with the concurrent relationship of the respondent. By comparing these two data domains, the impact of parallel events such as the relationships over the health of the respondent can be discovered. Such as, if a person were having more than one relationship at the same time then he/she would be more prone to HIV than others. Besides, by comparing Socio-Demographics survey to Health survey the impact of respondent's economic condition on health can be comprehended. By comparing multiple surveys, these important contexts regarding the causes of HIV can be identified.

## Discussion

The TRHC application allows collection of a large amount of data in the form of a formative qualitative-quantitative study. The main purpose of the TRHC study is to identify the reason behind older South Africans' performing or not performing an HIV test. Having information on individual characteristics and risk linked to time provides insights into what events occurred prior to a test or how those who test and do not test differ. It also allows assessment of how the risk histories can impact testing decisions at a particular moment and over time. Research focused on the risk of HIV acquisition among older persons are limited (41, 43, 45). The reason behind this is the cumbersome data collection approach, as well as less focus on older persons. Therefore, the TRHC application can play a vital role to facilitate the identification of the reasons behind the risk and testing for HIV among older South Africans by making the data collection process automated.

The fold-out paper grid (30, 50, 51) of TRHC had months in the top row and various life domains on the bottom-left side. Because of the potentially protracted and distal nature of older persons' risk profiles, TRHC was designed to include a 10-year retrospective period. While the TRHC paper fold-out was able to collect important information on social, relational, and health risk data, this process was tedious when the survey was carried out with a large population. Using the paper fold-out calendar, requires skillful interviewers to be able to go back and forth, correctly recording answer codes along the grid as well as moving between the calendar and survey questions. Our application is able to collect this information easily and is also able to store it to an online data source. The application prompts when to move to survey questions and has pull down menus for response codes, reducing the burden on the interviewer. This will increase the usability of the survey for both the interviewer and the respondent.

In the pilot, a set of public reference points (e.g., June/July 2010-World Cup, August 2012-Marikana Massacre, December 2013-Mandela's death) were pre-printed on the calendar; fieldworkers added respondents' salient personal reference points at the beginning of the interview. In the application, some reference points such as World Cup, Marikana, etc. are provided. The reference points added to a particular timeline can be modified or added according to the respondents' preference which makes the system dynamic. As the interview is progressed, information from one domain can be cross-checked and resolve inconsistencies in other domains using the dual-window visualization feature (24).

During the field testing of the paper-grid, the fluid form of the interview allowed fieldworkers to ask participants about HIV testing when “risk” was mentioned in any domain. Detailed margin comments and debriefing notes provided very valuable context to the data collected and allowed for an important in-depth understanding. The application's ability to automate and provide sufficient space for comments connected to any cell allows both for more plentiful details related to any incident (whereas space was limited on the paper calendar) and simplifies the data collection process.

The application was developed to ease the interview process involved in the TRHC study and thus the main interface was developed for the interviewer. The current application allows the collection of high-quality large-scale retrospective data by the interviewer. The future version of the application will include an interface for the interviewee to make the application more user friendly. In future, a usability evaluation of the application will be performed in order to test the compatibility of the application.

Although the application allows to collect large-scale qualitative-quantitative data, as with all retrospective studies, the data collected in this process depends on the memory of the respondent. While the instrument aims to collect detailed information on each sexual partner, respondents having multiple sequential or concurrent partners may not be aware of or remember all of the demographic details of their partners. Whatever details are remembered will be captured, but the respondent can always report that they do not know or do not remember these details. In formative research for this tool, very few older persons reported having more than 4 sexual partners in the previous 5 years, and even fewer reported that any of those partners were sex workers, so while these details may be more challenging for respondents to remember, their occurrence is infrequent. Similarly, issues like chemsex—whether related to drug or alcohol use—are less frequent in this population than they may be in younger populations, so this was not a significant consideration for building into the application. If the TRHC is adapted for younger populations, formative research will be needed to ensure that issues like these are addressed.

## Conclusion

The TRHC pilot study showed that older Africans were able to provide information about their social life, sexual relationship and health status, and which components changed over time and which were static; but the mechanism for collecting the data was cumbersome. The creation of a tablet application for facilitating higher quality data collection will allow us to collect these data in a larger population more efficiently in order to reveal key intervention points related to HIV testing and risk of the older South Africans.

The application makes the TRHC study suitable to be conducted at a larger scale by making the data collection process automated. In future, usability tests will be performed to refine the prototype in order to make the application more user-friendly so that maximum participation of respondents is achieved. Besides, this application can also be modified so that it can be customized in order to perform the TRHC study with other African populations such as younger generation to collect retrospective data in a broader range.

## Data Availability Statement

The original contributions presented in the study are included in the article/Supplementary Material, further inquiries can be directed to the corresponding author/s.

## Ethics Statement

Ethical review and approval was not required for the study on human participants in accordance with the local legislation and institutional requirements. The patients/participants provided their written informed consent to participate in this study. Written informed consent was obtained from the individual(s) for the publication of any potentially identifiable images or data included in this article.

## Author Contributions

AM developed the concept of the project as well as provided resources for the execution of the project and also supervised the whole project and reviewed and edited the manuscript. The methodology the project along with software execution was executed by DP and also developed the original draft of the manuscript and reviewed and edited it. LK and ES contributed to the original draft writing of the manuscript. ES provided data and resources to the project and reviewed the manuscript. All authors contributed to the article and approved the submitted version.

## Author Disclaimer

The context of this paper is solely the responsibility of the authors and does not necessarily represent the official views of the National Institute of Health.

## Conflict of Interest

The authors declare that the research was conducted in the absence of any commercial or financial relationships that could be construed as a potential conflict of interest.

## Publisher's Note

All claims expressed in this article are solely those of the authors and do not necessarily represent those of their affiliated organizations, or those of the publisher, the editors and the reviewers. Any product that may be evaluated in this article, or claim that may be made by its manufacturer, is not guaranteed or endorsed by the publisher.
