# Supplementary material for: Development of a Tablet Computer Application for HIV Testing and Risk History Calendar for Use With Older Africans
Source: Front Reprod Health. 2021 Dec 1;3:671747. doi: 10.3389/frph.2021.671747 (PMC9580772; doi:10.3389/frph.2021.671747)
Supplement: Supplementary file 1 [file Data_Sheet_1.docx]

Supplementary Material

# Supplementary Figures and Tables

## Supplementary Figures


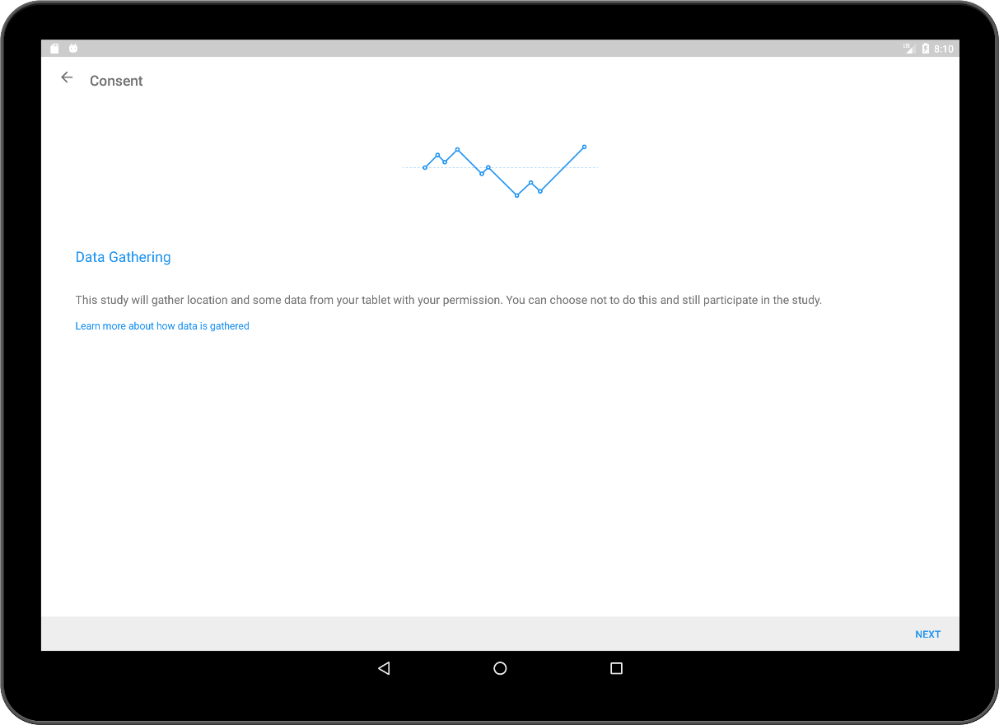


**Supplementary Figure 1.** Consent Activity (Data Gathering Details).


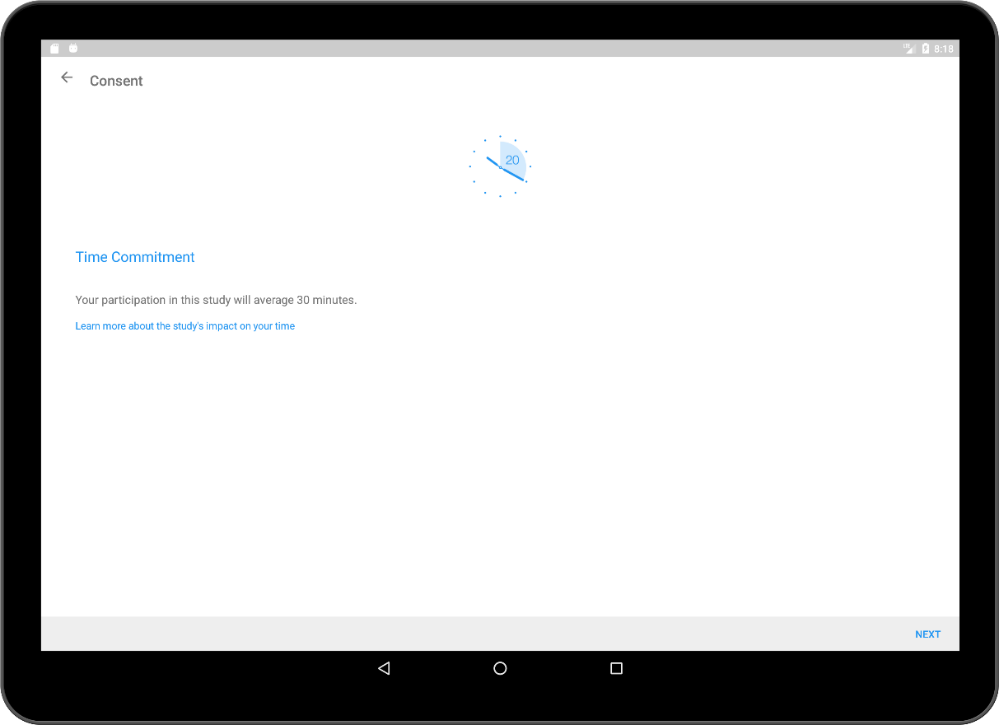


**Supplementary Figure 2.** Consent Activity (Time Requirement).


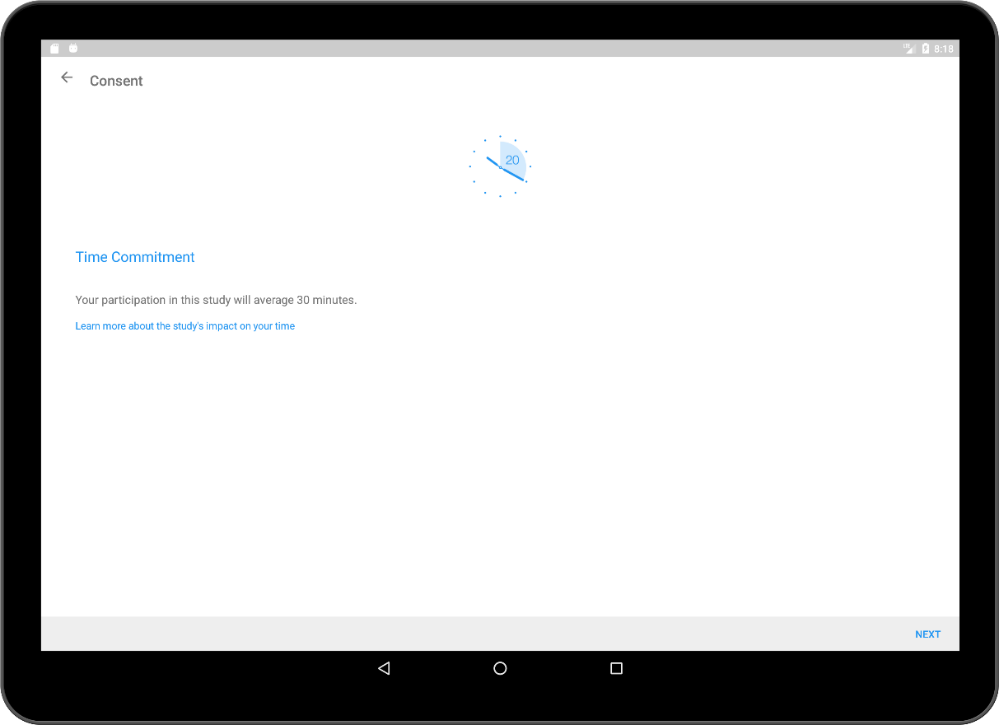


**Supplementary Figure 3.** Consent Activity (Signature).

## Supplementary Tables

Supplementary Table 1| Questionnaire related to HIV-testing behavior (generic)

| **HIV Questionnaire** | | |
| --- | --- | --- |
| **SI** | Questions | **Options** |
| 1. | How many times have you tested for HIV in your lifetime? | _________ Number  77 NEVER TESTED if 77, 88 or 99 - Trigger question to ask WHY NEVER TESTED  88 Don’t know  99 No response |
| 2. | When did you first test for HIV? | ________ MONTH ________ YEAR  88 Don’t know  99 No response |
| 3. | About how many times / how often did you test before First Year of Calendar? | _________ Number OR  55 Every few months  66 Annually/yearly  77 Every couple of years  88 Don’t know  99 No response |

Supplementary Table 2| Questionnaire related to individual HIV-testing behavior

| **Pop-Up Window for HIV Testing** | | |
| --- | --- | --- |
| **SI** | Questions | **Options** |
| 4. | Were you tested on your own initiative or because you were offered a test by a health worker at a facility or at home? | 1 Tested on own initiative  2 Offered test by health provider at a health facility  3 Offered test by health worker at home  4 Other (specify) _______  88 Don’t know  99 No response |
| 5. | What was the result of this HIV test? | 1 HIV pos  2 HIV neg  88 Don’t know  99 No response |
| 6. | Where did this HIV test take place? | 1 HIV public clinic  2 OPD public clinic  3 Private health facility  4 Self-tested at home  5 Mobile/pop-up testing  6 Occupational health/work testing  7 Other (specify) _____  88 Don’t know  99 No response |
| 7. | What was your motivation for this HIV test? | 1 Respondent wanted to know serostatus  2 Partner's sexual behavior  3 Partner told you to get tested  4 Partner ill or died  5 Partner HIV+  6 Child ill or died  7 Own sexual behavior  8 Taking care of PLWH  9 Symptoms made you or health provider think you might have HIV  10 Hospitalization for another reason  11 Being testing for TB  12 PMTCT  13 Provider said it was a routine part of care  14 future plans-marriage  15 Future plans-having children  16 Planning for future  17 Offered test at home  18 Other (specify)_____  88 Don't know  99 No response |
| 8. | At the time of this test, was there anyone in your family who was living with HIV? | 0 No  1 Yes, my partner  2 Yes, one or more of children/grandchildren  3 Yes, sibling  4 Yes, other member of my family  88 Don't know  99 No response |
| 9. | At the time of this test, had anyone in your family died, related to HIV/AIDS? | 0 No  1 Yes, my partner  2 Yes, one or more of children/grandchildren  3 Yes, sibling  4 Yes, other member of my family  88 Don't know  99 No response |
| 10. | Did you have a sexual partner at the time of this test? | 0 No  1 Yes |
| 11. | Did anyone go with you when you tested this time? | 0 No  1 Yes, my partner  2 Yes, other kin  3 Yes, friend  88 Don’t know  99 No response |
| 12. | Did the person who went with you when you to test also get tested? | 1 HIV pos  2 HIV neg  88 Don’t know  99 No response |
| 13. | What was the result of that person’s test? | 0 No one  1 Spouse/partner  2 Other partners  3 Adult child/ren  4 Grandchild/ren  5 Other kin  6 Boss/Workmates  7 Neighbors/ community  8 Pastor/church goers  9 Other (specify) ____  88 Don’t know  99 No response |
| 14. | Who did you disclose to about YOUR test this time? | _______ MONTH  88 Don’t Know  99 No Response  _______ YEAR  88 Don’t Know  99 No Response |
| 15. | Were you linked to care after you learned your results? | 0 No, I have never been linked to care  1 No, but was linked to care at a later time  2 Yes, started on ART  3 Yes, but not started on ART  88 Don’t know  99 No response |
| 16. | When did you start on ART? | 0 Not yet on ART  _____ Month  88 Don’t know  99 No Response  _____ Year  88 Don’t know  99 No Response |
| 17. | Are you still taking ART? | 0 No  1 Yes |
| 18. | How do you usually get your ART? | 1 I go pick them up myself at the clinic  2 I pick them up myself from a Central Dispensing Unit office/setting  3 Someone picks them up for me  4 Someone delivers them to me  5 Other (Specify) ______________ |
| 19. | How often do you receive /pick up your ART? | 1 Every month  2 Every 2 months  3 Every 3 months  Other (specify) _________  88 Don’t know  99 No Response |
| 20. | Have you ever experienced a stockout for your ART? | 0 No, never  1 Infrequently, but has happened  2 Often  3 Every time  88 Don’t know  99 No Response |
| 21. | In the last month have you ever missed taking a dose? | 0 No, never  1 Infrequently, but has happened  2 Often  88 Don’t know  99 No Response |
| 22. | In the past year, have you ever missed an HIV/ART appointment/club? | 0 No, never  1 Infrequently, but has happened  2 Often  88 Don’t know  99 No Response |
